# Chronic Effect of a Cafeteria Diet and Intensity of Resistance Training on the Circulating Lysophospholipidome in Young Rats

**DOI:** 10.3390/metabo11080471

**Published:** 2021-07-22

**Authors:** Susana Suárez-García, Antoni Caimari, Josep M. del Bas, Jaume Lalanza, Rosa M. Escorihuela, Manuel Suárez, Cristina Torres-Fuentes, Lluís Arola

**Affiliations:** 1 Nutrigenomics Research Group, Departament de Bioquímica i Biotecnologia, Universitat Rovira i Virgili, 43007 Tarragona, Spain; susana.suarez@urv.cat (S.S.-G.); cristina.torres@urv.cat (C.T.-F.); lluis.arola@urv.cat (L.A.); 2 Technological Unit of Nutrition and Health, EURECAT-Technological Center of Catalonia, 43204 Reus, Spain; antoni.caimari@eurecat.org (A.C.); josep.delbas@eurecat.org (J.M.d.B.); 3 Institut de Neurociències, Departament de Psiquiatria i Medicina Legal, Universitat Autònoma de Barcelona, 08193 Barcelona, Spain; jaume.lalanza@uab.cat (J.L.); rosamaria.escorihuela@uab.cat (R.M.E.)

**Keywords:** lysoglycerophospholipids, metabolic syndrome, cafeteria diet, resistance training

## Abstract

The daily practice of physical exercise and a balanced diet are recommended to prevent metabolic syndrome (MetS). As MetS is a multifactorial disorder associated with the development of serious diseases, the advancement of comprehensive biomarkers could aid in an accurate diagnosis. In this regard, it is known that gut microbiota is altered in MetS, and especially, lipid metabolites species are highly modified, thus emerging as potential biomarkers. In preliminary studies, we observed that alterations in serum lysoglycerophospholipids (Lyso-PLs) were shared between animals with diet-induced MetS and those performing resistance exercises assiduously. Therefore, our objective was the targeted determination of the lysophospholipidome in young rats fed a standard (ST) or a cafeteria diet (CAF) and submitted to different training intensities to evaluate its potential as a biomarker of a detrimental lifestyle. Targeted metabolomics focused on lysophosphatidylcholines (Lyso-PCs) and lysophosphatidylethanolamines (Lyso-PEs) and multivariate statistics were used to achieve an integral understanding. Chronic intake of CAF altered the serological levels of both lipid subclasses. Twenty-two Lyso-PLs were significantly altered by CAF, from which we selected Lyso-PCs (14:0), (17:1) and (20:2) and Lyso-PEs (18:2) and (18:3) as they were enough to achieve an optimal prediction. The main effect of physical training was decreased Lyso-PEs levels with disparities among training intensities for each diet. We concluded that an examination of the lysophospholipidome reveals the general state of the metabolome in young female rats, especially due to intake of an MetS-inducing diet, thus highlighting the importance of this family of compounds in lipid disorders.

## 1. Introduction

Metabolic syndrome (MetS) represents a serious public health problem due to its increasing prevalence, which can reach around 80% in some regions [1]. The incidence depends on multiple factors including age, gender, ethnicity, and social status, which includes cultural and economic factors. Among others, the composition of the diet, overnutrition, gut microbiota dysbiosis and level of physical activity can constitute important risk factors for MetS and its components [1,2]. However, a detrimental lifestyle can be easily corrected if diagnosed properly, contributing to the prevention and treatment of metabolic disorders, especially in young people [3,4]. The advancement of comprehensive biomarkers could provide an accurate diagnosis. Among these biomarkers, those affected by gut microbiota are of special interest as these bacteria significantly influence the development of MetS [5]. Lipid metabolites are one of the most influenced by gut bacteria [6]. Indeed, we have recently shown that gut bacteria significantly affect lipid mediators metabolites, known as oxylipins, and that the profile of these metabolites could be used as new obesity markers [7]. In addition, changes in the gut microbiota in an obesity model have been associated with alterations in the lysophospholipid species (Lyso-PLs) [8]. Therefore, the use of lipid metabolite profiles as MetS biomarkers may have remarkable potential. 

In previous studies, we have observed that feeding with a highly palatable cafeteria diet (CAF) induces a profound impact on the gut microbiota profile [7,9] as well as in the serum metabolome, impairing the lipid metabolism and inflammatory response and leading to the development of MetS in rats [10]. In particular, CAF feeding increased the body weight gain and retroperitoneal white adipose tissue (RWAT) depot mass and induced the advance of hypertriglyceridemia, hyperleptinemia, hyperglycemia and insulin resistance. We also observed that the practice of daily exercise had a minor impact on the animal metabolome by reducing the serum levels of different lipid species. Regular training exerted a therapeutic effect on MetS by means of a reduction in the RWAT weight, leptin/adiponectin ratio in serum and triglycerides levels [10,11]. Among lipids, extracellular glycerophospholipids, including lysophosphatidylcholines (Lyso-PCs) and lysophosphatidylethanolamines (Lyso-PEs) were the most altered metabolites. These are deacylated forms of phospholipids that can be synthesized de novo from glycerol-3-phosphate and fatty acyl-CoA or through the enzymatic hydrolysis of phosphatidycholines and phosphatidylethanolamines, respectively, under phospholipase activity [12]. Both classes of Lyso-PLs are considered bioactive molecules with signaling and regulatory capacities [13]. With the progress in omics technologies in the last years, imbalances in Lyso-PL levels have been closely related to atherosclerosis [14,15], nonalcoholic fatty liver diseases [16,17,18] and childhood and adult obesity [19,20,21,22]. In this context, Pietiläinen et al. demonstrated in young adult twins that circulating Lyso-PC content was associated with acquired obesity independently of genetic factors [23]. In this regard, we further observed that the alterations in non-obese dyslipidemic animals were due to the endogenous metabolism rather than diet [24]. It is also known that dietary habits conducive to treating MetS have compensatory effects on the plasma levels of both classes of Lyso-PLs [25,26,27]; however, there is very little research on endurance exercise and the influence of different training intensities on the Lyso-PL-related metabolome associated to MetS.

Therefore, the main objective of the present investigation was the detailed evaluation of Lyso-PL levels in the serum of young rats fed standard chow (ST) or CAF and submitted to different intensities of aerobic training performed voluntarily on a treadmill without inclination. Thus, we aimed to assess the potential of Lyso-PLs as circulating biomarkers of lipid disorders in youth by using a targeted metabolomics analysis.

## 2. Results

### 2.1. Determination of the Circulating Levels of Lyso-PLs

As we have previously published, a CAF diet for 8 weeks triggered the development of MetS in animals [11]. In particular, at the end of the study all CAF-fed animals were overweight compared with their ST-fed counterparts. In addition, body weight gain and retroperitoneal white adipose tissue (RWAT) depot mass were also higher in the CAF animals, as well as the levels of triglycerides, leptin, glycemia and insulin. Therefore, the impact of the diets on the development of MetS was clearly confirmed. Focusing on the aim of the study, targeted metabolomics analysis of thirty-one Lyso-PLs in rat serum was conducted in the experimental groups showed in the Supplementary Materials, Figure S1. The circulating levels, expressed as micro molarity (µM), are reported in Table 1. In general, the serum concentrations of Lyso-PEs, with maximum levels of barely a dozen µM, were lower than Lyso-PCs, among which those with saturated acyl chains of 16 and 18 carbons reached levels of several hundred µM. Other abundant Lyso-PLs in rat serum were Lyso-PCs (20:4) and (18:2). A similar pattern of concentrations was followed within Lyso-PEs.

**Figure 1:**
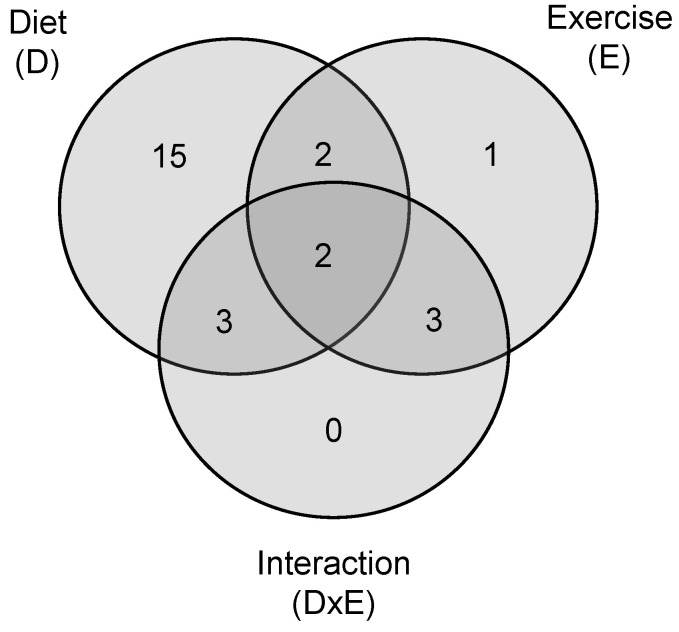
Venn diagram showing the number of serum Lyso-PLs for each experimental factor. Data were analyzed using two-way ANOVA. D: the effect of diet; E: the effect of exercise; DxE: the interaction between the two main factors (*p* < 0.05). The areas where the circles overlap show the number of significant lyso forms shared by the factors. The flowchart of the experimental design is showed in Supplementary Materials, Figure S1.

Based on the comparison of the six groups by two-way ANOVA, the lifestyle intervention induced significant modifications in the levels of the majority of Lyso-PLs (Figure 1), specifically, in twenty-six of them. As Table 1 shows, these differences were present in both Lyso-PCs and Lyso-PEs and more than 70% were due to the type of feeding. Thus, while the circulating levels of Lyso-PCs (14:0), (16:1), (17:1), (18:1), (20:3), (20:5), (22:5) and Lyso-PEs (16:1), (18:1), (20:3), (20:4), (22:5) were increased; the levels of Lyso-PCs (15:0), (17:0), (18:2), (18:3), (20:0), (20:1), (20:2) and Lyso-PEs (18:2), (18:3), (20:2) decreased in response to CAF intake. As a result, when animals were fed a CAF diet, the levels of Lyso-PLs with different head groups but identical acyl chains typically followed comparable trends.

On the other hand, the effect of physical training was prevalent in Lyso-PEs, influencing the levels of almost half of the molecular species, while only 10% of the Lyso-PCs were altered (*p* < 0.050, two-way ANOVA). Along with diet, the levels of Lyso-PEs (18:1) and (20:4) were affected by exercise. The use of the treadmill counteracted the high levels induced by CAF intake, mainly in the group that was running at low speed (TML-CAF), in which significant differences in the levels of both metabolites were observed compared to the CON-CAF group (Table 1). Circulating Lyso-PE (16:1), which was also influenced by diet and exercise, behaved similarly to the others but the drop in levels was observed in both trained groups of CAF-fed animals as residual responses (*p* < 0.020, Student’s t-test). No significant changes in these Lyso-PEs were found among ST-fed groups. In fact, Lyso-PEs (16:1) and (20:4) were also significantly affected by the interaction of the two experimental factors. Instead, Lyso-PC (16:0) was exclusively influenced by exercise, which led to a general decrease in the circulating levels of animals fed both diets. According to the post hoc analysis, other decreases in Lyso-PL levels as a consequence of exercise were detected in Lyso-PEs (16:0), (18:0) and (22:6) and, curiously, were also found between CON-CAF and TML-CAF groups.

Based on these results, the chronic intake of a MetS-inducing diet elicited drastic changes in Lyso-PL levels that involved the two lipid subclasses, Lyso-PCs and Lyso-PEs, and that in many cases were dependent of the fatty acid structure. The effect of physical training was minor and particularly focused on Lyso-PEs, where it was able to steady the levels of some of them in CAF-fed rats, especially when they had performed exercise at a moderate intensity.

### 2.2. Multivariate Statistics Reveals Lyso-PLs Alteration Association with Cafeteria Diet

**Figure 2:**
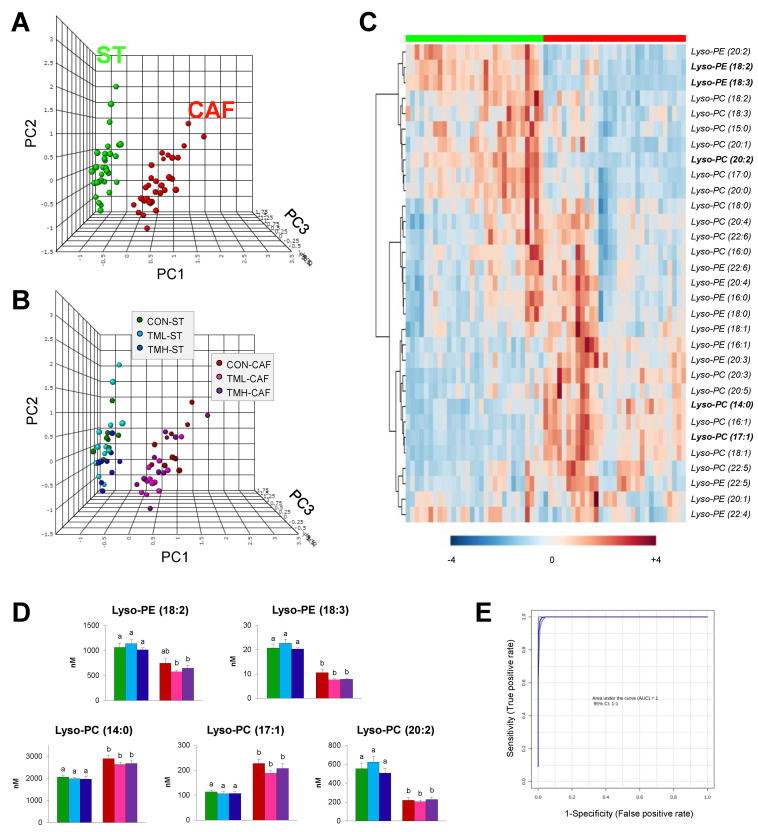
Identification of CAF-related biomarkers. (**A**) Three-dimensional score plot of principal component analysis (PCA) of the thirty-one Lyso-PLs quantified in rat serum. Animals fed standard chow (ST) are represented as green spots and animals fed cafeteria diet (CAF) as red spots. (**B**) Score plot shown on panel A with spots colored as ST or CAF-fed animals trained on a treadmill at different intensities (CON: 0; TML: 12; TMH: 17 m/min) for 2 months. (**C**) Heatmap plot of hierarchical clustering analysis of the Lyso-PL levels in each dietary group. Each row represents a molecular specie colored by its range-scaled abundance intensity. The scale from −4 (blue) to +4 (red) represents this normalized abundance in arbitrary units. (**D**) Circulating levels expressed on nano molarity (nM) of the best cafeteria diet-related biomarkers in the six groups of animals (*n* = 9–12). Mean values with different lowercase letters were significantly different (one-way ANOVA and Tukey or Dunnett’s T3 post hoc contrasts, *p* < 0.05). (**E**) Receiver operating characteristic (ROC) curve analysis using the five Lyso-PLs with the variable of highest importance in the discrimination of both dietary groups. The area under the curve (AUC) and the corresponding confidence interval (CI) were optimal.

A multivariate statistical analysis was performed in order to uncover the Lyso-PLs that best reflect the CAF-phenotype in rats (Figure 2).

In a first step, PCA models including all the identified Lyso-PLs were used to compare the importance of the effect of each experimental factor, diet and physical activity on their levels. As can be seen, it was possible to distinguish a clear separation between animals according to the diet administered, without resorting to a supervised method of discriminant analysis (Figure 2A). Thus, ~76% of the variance was explained when the scores of the first three principal components were represented. However, the effect of the physical exercise was much less and when the individual scores were colored according to diet and training intensity, discriminating between the three exercise levels was difficult (Figure 2B). The heatmap from the hierarchical clustering analysis showed two distinct patterns in Lyso-PL levels according to the diet administered (Figure 2C).

In the differentiation of the dietary groups, the five Lyso-PLs with the greatest weight were Lyso-PE (18:3) > Lyso-PC (20:2) > Lyso-PC (14:0) > Lyso-PC (17:1) > Lyso-PE (18:2) (Figure 2D). The serum levels of both Lyso-PEs were decreased in CAF-fed groups, whereas Lyso-PC followed different trends. Further validation of the CAF-related biomarker was carried out by plotting ROC curves using a different number of variables under consideration. The results showed that the five Lyso-PLs referred to above were sufficient to achieve an optimal prediction, based on the peak values of the area under the curve and its corresponding confidence interval (Figure 2E). This summarized model presented a null error rate in classifying the animals according to the diet. These findings suggest that Lyso-PLs are excellent predictors of the reiterated consumption of CAF in rat serum, even when animals were subjected to low or high intensities of endurance training.

### 2.3. Lyso-PLs as Poor Indicators of the Practice of Daily Exercise

**Figure 3:**
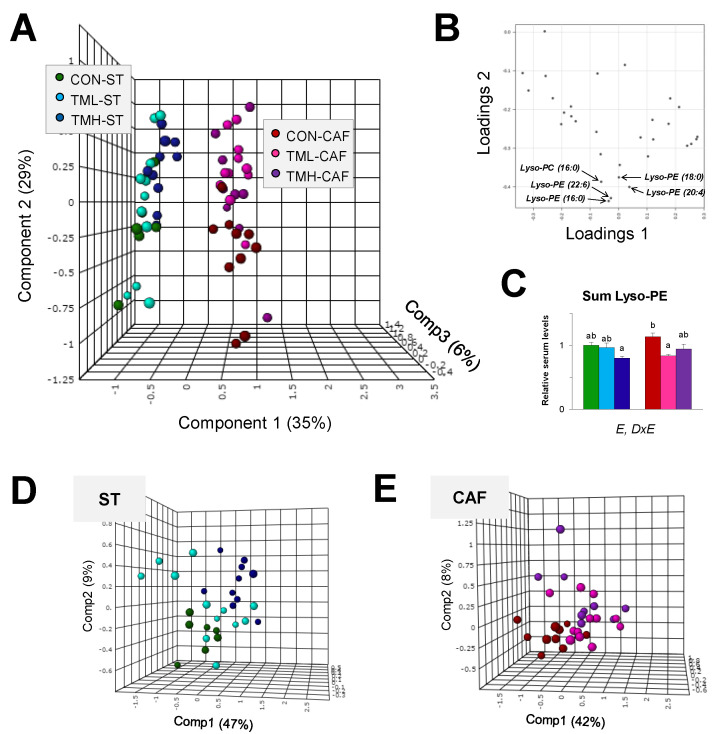
Identification of biomarkers of the daily practice of physical exercise. (**A**) Three-dimensional scores plot of partial least squares discriminant analysis of the thirty-one Lyso-PLs detected in rat serum. Each spot represents an animal colored according to both diet (ST or CAF) and intensity of training performed on a treadmill (CON: 0; TML: 12; TMH: 17 m/min) for 2 months. (**B**) Two-dimensional loadings plot showing the main underlying Lyso-PLs responsible for the discrimination between trained groups. (**C**) Total sum of the serum Lyso-PE levels (thirteen molecular species). The data are presented as means ± SEM (*n* = 9–12). The statistical comparison among the animal groups was conducted using two- and one-way ANOVA. E: the effect of exercise; DxE: the interaction between diet and exercise (two-way ANOVA, *p* < 0.05). abc: Mean values with different lowercase letters were significantly different (one-way ANOVA and Tukey post hoc contrast, *p* < 0.05). Separate score plot for ST (**D**) and CAF (**E**) groups showing the slight influence of exercise on the Lyso-PL metabolome.

Once the prevalent effect of diet was determined, we proceeded to examine the influence of physical exercise on the Lyso-PL-related metabolome (Figure 3).

On this occasion, it was necessary to use a supervised form of discriminant analysis for the separation of the six animal classes in a score plot explaining ~70% of co-variance (Figure 3A). The extent of fit of the PLS-DA model to the metabolomics data, represented by R2, was 0.84. However, the internal cross-validation indicated that the prediction accuracy was only 40%. Even though the scores diagram showed the discrimination of the animals mainly in dietary classes along the first component, it was also noticeable that trained groups formed distinct clusters regarding sedentary animals along the second component of PLS-DA. In relation to this, the resultant loadings plot indicated that Lyso-PEs (16:0), (18:0), (20:4) and (22:6) and Lyso-PC (16:0) were the physical activity-related metabolites of greatest importance in the classification of the sedentary groups (Figure 3B). Therefore, Lyso-PEs were better biomarkers of aerobic training than Lyso-PCs. In fact, the whole Lyso-PE family showed a global involvement in resistance exercise-related metabolism (*p* = 0.001, two-way ANOVA) with general behavior similar to that described above for individual Lyso-PEs (Figure 3C). This circulating trend in the Lyso-PE levels was different in relation to diet (*p* = 0.015, two-way ANOVA). Based on the individual scores plot of ST (Figure 3D) and CAF-fed rats (Figure 3E), the effect of exercise on the Lyso-PL-related metabolome, although very slight, seemed to be gradual and exclusively in the ST-fed groups. Thus, the separation from the sedentary animals was larger for the most intensely trained group (TMH-ST) than for the middle condition, which showed a higher degree of data dispersion (Figure 3D). However, even having eliminated the predominant influence of diet, the permutation tests of the two prediction models were not significant and the error rates for classifying trained animals within dietary groups were over 50%.

Therefore, although Lyso-PLs, particularly Lyso-PEs, were shown to be involved in the biological effects of exercise on trained rats, the influence that diet has on the circulating Lyso-PL-related metabolome was so strong that daily resistance training carried out at the analyzed intensities was only lightly captured in the serum Lyso-PL levels. In addition, differences between exercise intensities were also observed and they are dependent on the type of feeding.

## 3. Discussion

In the present investigation, we have shown that the administration of CAF to weaning rats for 8 weeks induced several alterations in the Lyso-PL-related metabolome. This result confirms those previously found in studies using non-targeted metabolomics [10]. Moreover, this study provides accurate and detailed information regarding the relevance of the chronic intake of a MetS-inducing diet and physical exercise on the circulating levels of Lyso-PLs in rats. Approximately 80% of the Lyso-PCs targeted and evaluated in serum were implicated, and also more than 60% of the Lyso-PEs evaluated were also involved (Table 1). These results are in accordance with other studies performed in CAF-fed rats with hypertriglyceridemia [28], as well as in different rodent species fed diets high in fat [29,30] and subjects with obesity [20,22,30]. All of them reported several dysregulations in Lyso-PL levels, which was more pronounced in plasma than tissues in the case of animals. These Lyso-PLs are considered signaling molecules that mediate inflammatory processes. In this regard, some authors have associated the promotion of inflammatory processes, linked to alterations in the circulating levels of fatty acids and bile acids, with changes in the composition of the intestinal microbiota within the framework of obesity and metabolic syndrome [31]. All of these results agree with the findings observed in this study and our previous results [10].

In order to simplify the predictive model and facilitate the potential use of these lipid species as biomarkers in routine analysis, we decided to select those compounds that allowed us to distinguish between dietary groups without losing reliability. Thus, the array was limited to five metabolites that as a whole were enough to achieve an ideal prediction, regardless of whether the animals performed physical activity (Figure 2D,E). These key metabolites are the Lyso-PCs (14:0), (17:1) and (20:2) and the Lyso-PEs (18:2) and (18:3). Consistent with our observations, while decreased plasma levels of Lyso-PE (18:2) were detected in rodents with diet-induced obesity [29], a similar trend was identified in the serum of obese children when linoleic acid was associated with choline as a polar group [20]. Another important Lyso-PL, whose levels decreased with the chronic ingestion of a MetS-inducing diet was Lyso-PC (20:2). Interestingly, the circulating levels of this metabolite decreased in rats and patients with obesity [28,30], suggesting no difference between these species. Serum increments of Lyso-PC (14:0) have also been described in overweight subjects in comparison with lean subjects [19], and in another study, this was the metabolite that best discriminated among dyslipidemic and healthy men [32]. All these results agree with those obtained in the present work and support the potential of this set of metabolites as accurate biomarkers in MetS.

On the other hand, although less significant, modifications in the levels of some Lyso-PL were also associated with the practice of exercise in trained rats. However, dietary habits promote a deep sharp change in the circulating Lyso-PL-related metabolome, while the consequences of the resistance training are masked and slightly reflected on a global level. Notably, these results are consistent with other investigations conducted on trained rodents in which differential patterns in metabolic spectra were identified but these were unable to fully reverse the detrimental effects of a diet rich in fat [33,34]. In the present study, Lyso-PE levels were the Lyso-PL class primarily affected by exercise, especially in animals with MetS in which moderate intensity was the most influential (Table 1 and Figure 3B,C). The Lyso-PL-lowering effect observed in serum could be caused by the increased accumulation of these lipid metabolites in tissues. In this regards, increments in several Lyso-PLs have been reported in muscle, and particularly in liver of trained rodents with regard to sedentary animals [35,36]. Intracellular autophagy could contribute to the alteration in the circulating Lyso-PL profile after physical training, and thereby could explain the association between these molecules and metabolic diseases. Furthermore, it has been demonstrated that Lyso-PC (16:0), one of the few Lyso-PCs affected by exercise in our study, upregulates the expression of PPARα target genes in hepatocytes [37]. The levels of this particular species was decreased in the serum of trained rats regardless of diet and it has also been described as a key compound in the recognition of mice that perform aerobic exercise frequently [38]. Interestingly, other authors have shown that some diets with hypocholesterolemic properties are also able to restrain the circulating level of Lyso-PC (16:0), as well as those of Lyso-PEs (16:0), (18:0) and (20:4) [25,27].

In conclusion, the examination of the lysophospholipidome reveals the general state of the metabolome due to the intake of a MetS-inducing diet and the practice of exercise by using a targeted approach, thus highlighting the importance of this family of compounds in lipid disorders. Based on these results, we conclude that the analysis of the plasma lysophospholipidome could be a good strategy to use in the diagnosis of dyslipidemia-related diseases. Specifically, Lyso-PCs (14:0), (17:1) and (20:2) and the Lyso-PEs (18:2) and (18:3) may have great potential as key biomarkers of metabolic disorders.

## 4. Materials and Methods

### 4.1. Ethics Statement

The animal protocol was approved by the Generalitat de Catalunya. All of the procedures were performed following the “Principles of Laboratory Animal Care” and according to the European Communities Council Directive regarding the protection of experimental animals (86/609/EEC).

### 4.2. Animals

The experimental animals were the same as those used in our previous work [10]. Briefly, young female Sprague Dawley rats, weaned at 21–23 days of age, and weighing 62 ± 2 g were divided into 6 groups (*n* = 9–12 per group) with comparable body weights. The selection of female animals was based on our previous studies in which female animals were more active in the voluntary wheel running and presented higher tolerance to physical training intensities compared to males [39]. An outline of the experimental design is showed in the Supplementary Materials, Figure S1. For 8 weeks, the animals were fed ad libitum either ST (Harlan, Barcelona, Spain) or CAF. ST had a calorie breakdown of 58% carbohydrates, 24% protein and 18% fat, whereas the CAF diet had 49% carbohydrates, 10% protein and 41% fat. The CAF diet included the following components (quantity per rat/day): ST (6 ± 10 g), sweet roll (8 ± 10 g), bacon (8 ± 12 g), biscuits with pate (12 ± 15 g) or cream cheese (10 ± 12 g), carrot (6 ± 9 g) and milk with sugar (220 g/L; 50 mL). The animals consumed diet and tap water ad libitum throughout the experiment, and CAF was renewed daily. Both dietary groups were further submitted to periodic training on a treadmill at different intensities (CON: 0; TML: 12; TMH: 17 m/min). The training sessions were organized 5 days per week and extended for 30 min. The animals were fasted overnight (12 h) and alternately sacrificed by beheading. Total blood was collected and serum was obtained by centrifugation (2000× *g*, 15 min, 4 °C). Samples were conserved at −80 °C until metabolite extraction and LC-MS/MS analysis. Body weight, food intake and biometric parameters of these animals can be seen in our previous publications [10,11].

### 4.3. Chemicals

The mobile phases used for the chromatographic separation of Lyso-PLs were prepared with methanol (MeOH) (Scharlab, Barcelona, Spain), acetonitrile (Millipore, Darmstadt, Germany), isopropanol and 7.5 M ammonium acetate solution (Sigma-Aldrich, St. Louis, MO, USA). All of these were of the highest grade commercially available. Ultrapure water was obtained from a Milli-Q advantage A10 system (Madrid, Spain).

The standards using in the LC-MS/MS analysis of Lyso-PLs were 1-tridecanoyl-sn-glycero-3-phosphocoline, Lyso-PC (13:0); 1-palmitoyl-sn-glycero-3-phosphocoline, Lyso-PC (16:0); 1-stearoyl-sn-glycero-3-phosphocoline, Lyso-PC (18:0); 1-arachidoyl-sn-glycero-3-phosphocoline, Lyso-PC (20:0); 1-palmitoyl-sn-glycero-3-phosphoethanolamine, Lyso-PE (16:0); 1-stearoyl-sn-glycero-3-phosphoethanolamine, Lyso-PE (18:0); and 1-oleoyl-sn-glycero-3-phosphoethanolamine, Lyso-PE (18:1). All of these were over 99% purity and were purchased from Avanti Polar Lipids (Birmingham, AL, USA). A solution containing chloroform (Sigma-Aldrich, St. Louis, MO, USA) was used for the dilution of standards.

Butylated hydroxytoluene (BHT, Sigma-Aldrich, St. Louis, MO, USA) was added to MeOH to avoid metabolite oxidation during sample extraction.

### 4.4. Preparation of Standard Curves

Lyso-PLs were individually dissolved in MeOH/chloroform/water (65:35:8 *v*/*v*/*v*) at 2 mg/mL and preserved in dark-glass vials at −20 °C. The day of the LC-MS/MS analysis, mixed standard solutions with concentrations of 1, 10 and 100 mg/L were prepared using MeOH. Similar to the calibrators, Lyso-PC (13:0) was handled separately to be used as internal standard (IS).

The calibration curves were prepared by the addition of increasing amounts of the standard mixtures to constant final volumes of water/isopropanol/acetonitrile (4:3:3 *v*/*v*/*v*) in the presence of the IS. The concentration of the calibrators ranged from 0 to 5 mg/L, whereas the IS was added at a final concentration of 500 or 360 µg/L depending on the procedure of extraction. The preparations were extracted and subsequently analyzed by using the same procedure as the serum samples. The calibration curves were finally generated for each standard by plotting the peak abundance ratios (analyte/IS) versus the concentration ratios (analyte/IS) and fitting to a linear regression. Supplementary Materials, Figure S2 shows the standard curves used for the analysis of endogenous Lyso-PLs.

### 4.5. Sample Processing

**Figure 4:**
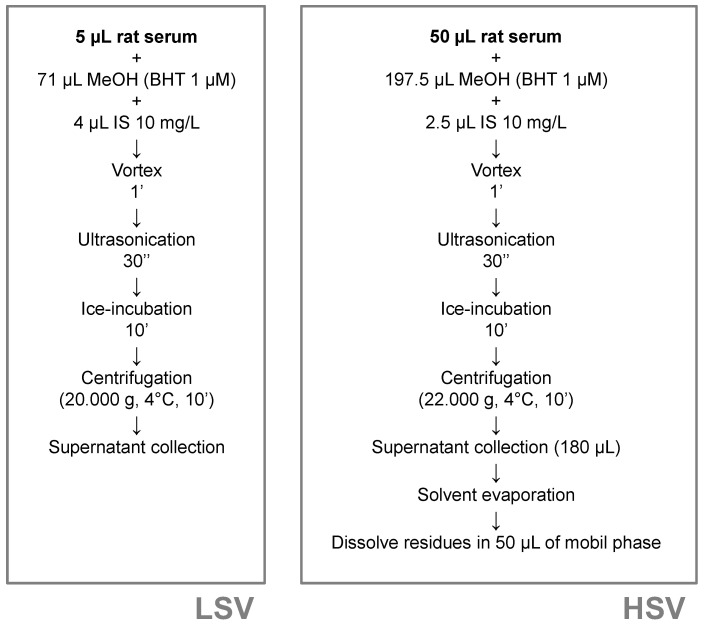
Extraction procedures for the exhaustive metabolomics analysis of Lyso-PLs.

Circulating Lyso-PLs were found in a wide range of concentrations. Due to that, two different procedures for serum processing were required in the present study. Both of them are based on liquid–liquid extractions. The first one was aimed at the analysis of the most abundant compounds, mainly Lyso-PCs, and needed only 5 µL of sample; so, we called it the “Low sample volume” (LSV) extraction. The second procedure assisted in the determination of serum Lyso-PEs and starts with a greater volume (50 µL) of sample, so it was called the “High sample volume” (HSV) method. Figure 4 shows an outline of the steps followed in each procedure.

### 4.6. Targeted Metabolomics Analysis of Lyso-PLs

In order to cover all the Lyso-PLs contained in the samples, a specific UHPLC- + ESI-MS/MS analysis was developed and validated using a set of samples from this study. Details and results from this validation are shown in our previous publication [40]. The chromatographic separation was performed in an UHPLC 1290 (Agilent Technologies, Palo Alto, CA, USA) composed of a degasser, a binary pump, and thermostatted autosampler and column compartments kept at 4 °C and 50 °C, respectively, during the analysis. The mobile phase consisted of water/isopropanol/acetonitrile/500 mM ammonium acetate (89:5:5:1 *v*/*v*/*v*/*v*) as solvent A and isopropanol/acetonitrile/500 mM ammonium acetate (50:49:1 *v*/*v*/*v*) as solvent B. Each sample (2 µL) was loaded into a 0.3 mL/min flow of 30% B passing through a reversed-phase column (Acquity UPLC BEH C8) with a particle size of 1.7 µm and 2.1 mm internal diameter ×150 mm length (Waters Corporation, Milford, MA, USA). After that, the analytes eluted along a continuous gradient to 75% B over 20 min. A pool of samples was used as quality control in order to monitor and control the analysis.

The tandem mass spectrometer analysis was achieved through positive electrospray ionization (+ESI) performed within a QqQ 6490, also from Agilent Technologies. The ionization source parameters were as follows: nebulizer gas (nitrogen) pressure, 25 psi; gas flow, 12 L/min at 240 °C; sheath gas flow, 12 L/min at 350 °C; capillary and nozzle voltages, 4.5 kV and 500 V, respectively; and fragmentor and cell accelerator voltages, 380 and 5 V, respectively. The selected scan mode was dynamic multiple reaction monitoring (MRM). Information about the transitions and optimal collision energies used for UHPLC- + ESI-MS/MS analysis is provided in Supplementary Materials, Table S1.

### 4.7. Statistical Analyses

#### 4.7.1. Univariate Analysis

The results are presented as the means ± standard errors (SEM) based on the indicated number of rats. The Shapiro–Wilk test was used for the assessment of the normality of the data. After that, differences among the six groups of rats were determined using two and one-way ANOVA. First, analysis based on two-way ANOVA was used to evaluate the main effects of the diet and the physical exercise and their interaction. When any of the effects was statistically significant, one-way ANOVA was used to determine the differences among means. The homoscedasticity between groups was assessed using Levene’s test. Tukey’s post hoc contrast was applied when the variances were similar, whereas Dunnett’s T3 post hoc contrast was applied if this assumption was not fulfilled. A two-tailed value of *p* < 0.05 was considered statistically significant. The univariate statistical analysis was performed with the Statistical Package for Social Sciences IBM Corp. Released 2010. IBM SPSS Statistics for Windows, Version 19.0. Armonk, NY, USA: IBM Corp.

#### 4.7.2. Multivariate Analysis

A multivariate statistical evaluation based on a combination of principal components analysis (PCA), partial least squares discriminant analysis (PLS-DA) and hierarchical clustering analysis was performed to determine the influence of diet and physical exercise on the Lyso-PL-related metabolome. Receiver operating characteristic (ROC) curves by the “leave one out” approach, permutation tests and random forest classification were also conducted to validate the multivariate biomarker. All analyses were performed after range scaling with the use of the software MetaboAnalyst (version 3.0) available online [41].

## Figures and Tables

**Table 1:** Lysophospholipid levels in serum of rats fed a standard chow (ST) or a cafeteria diet (CAF) and periodically trained on a treadmill at different intensities (CON: 0; TML: 12; TMH: 17 m/min).

Metabolite (µM)	CON-ST	TML-ST	TMH-ST	CON-CAF	TML-CAF	TMH-CAF	2-Way ANOVA
Lyso-PCs							
(14:0)	2.062 ± 0.059 ^a^	1.988 ± 0.064 ^a^	1.969 ± 0.113 ^a^	2.894 ± 0.143 ^b^	2.632 ± 0.108 ^b^	2.674 ± 0.129 ^b^	D
(15:0)	1.530 ± 0.075 ^a^	1.323 ± 0.057 ^a,b^	1.298 ± 0.064 ^a,b^	1.220 ± 0.044 ^b^	1.113 ± 0.038 ^b^	1.168 ± 0.051 ^b^	D, E
(16:0)	154.774 ± 0.740 ^a^	138.183 ± 7.787 ^a,b^	134.710 ± 4.210 ^b^	145.448 ± 3.447 ^a,b^	126.934 ± 4.947 ^b^	131.106 ± 7.666 ^a,b^	E
(16:1)	2.298 ± 0.135 ^a^	2.405 ± 0.176 ^a,c^	2.191 ± 0.122 ^a^	4.378 ± 0.326 ^b^	3.405 ± 0.127 ^b^	3.744 ± 0.342 ^b,c^	D, DxE
(17:0)	3.374 ± 0.209 ^a^	3.521 ± 0.278 ^a,b^	2.962 ± 0.207 ^ac^	2.517 ± 0.122 ^b,c^	2.289 ± 0.116 ^c^	2.195 ± 0.177 ^c^	D
(17:1)	0.113 ± 0.006 ^a^	0.106 ± 0.008 ^a^	0.107 ± 0.007 ^a^	0.227 ± 0.017 ^b^	0.188 ± 0.012 ^b^	0.207 ± 0.020 ^b^	D
(18:0)	223.830 ± 8.906	254.579 ± 16.940	220.397 ± 10.399	235.556 ± 12.324	211.205 ± 9.884	223.689 ± 10.308	
(18:1)	10.704 ± 0.282 ^a^	12.041 ± 1.212 ^a^	10.943 ± 0.652 ^a^	22.178 ± 1.427 ^b^	17.845 ± 1.032 ^b^	21.538 ± 1.940 ^b^	D, DxE
(18:2)	31.608 ± 1.393 ^a^	31.560 ± 2.673 ^a,b^	28.4467 ± 1.703 ^a,b^	23.611 ± 1.627 ^b,c^	21.097 ± 1.051 ^c^	24.427 ± 2.059 ^a,c^	D
(18:3)	0.471 ± 0.039	0.579 ± 0.074	0.512 ± 0.075	0.382 ± 0.049	0.318 ± 0.039	0.317 ± 0.048	D
(20:0)	0.504 ± 0.031 ^a^	0.530 ± 0.045 ^a^	0.434 ± 0.013 ^a,b^	0.308 ± 0.022 ^b,c^	0.271 ± 0.021 ^c^	0.276 ± 0.028 ^c^	D
(20:1)	1.425 ± 0.079 ^a^	1.441 ± 0.106 ^a^	1.122 ± 0.089 ^a,b^	0.888 ± 0.048 ^b,c^	0.763 ± 0.056 ^c^	1.109 ± 0.157 ^a,c^	D, DxE
(20:2)	0.552 ± 0.059 ^a^	0.622 ± 0.062 ^a^	0.506 ± 0.052 ^a^	0.219 ± 0.028 ^b^	0.203 ± 0.018 ^b^	0.227 ± 0.025 ^b^	D
(20:3)	0.757 ± 0.062 ^a,c^	0.803 ± 0.115 ^a,c^	0.722 ± 0.107 ^a^	1.724 ± 0.136 ^b^	1.647 ± 0.122 ^b^	1.814 ± 0.273 ^b,c^	D
(20:4)	53.596 ± 2.983	55.154 ± 5.681	46.297 ± 3.136	61.430 ± 2.813	51.069 ± 3.096	55.131 ± 4.538	
(20:5)	0.117 ± 0.027 ^a^	0.137 ± 0.029 ^a,b^	0.120 ± 0.033 ^a,b^	0.266 ± 0.035 ^b^	0.189 ± 0.025 ^a,b^	0.250 ± 0.074 ^a,b^	D
(22:5)	0.595 ± 0.128 ^a,b^	0.470 ± 0.064 ^a^	0.470 ± 0.047 ^a^	0.807 ± 0.059 ^b^	0.738 ± 0.110 ^a,b^	0.757 ± 0.141 ^a,b^	D
(22:6)	6.452 ± 0.675	6.622 ± 0.695	4.863 ± 0.244	6.357 ± 0.388	4.950 ± 0.415	5.253 ± 0.585	
Lyso-PEs							
(16:0)	4.730 ± 0.291 ^a^	4.484 ± 0.319 ^a,b^	3.510 ± 0.165 ^b^	4.923 ± 0.215 ^a^	3.749 ± 0.183 ^b^	4.241 ± 0.355 ^a,b^	E, DxE
(16:1)	0.027 ± 0.002 ^a^	0.030 ± 0.003 ^a,c^	0.028 ± 0.002 ^a^	0.058 ± 0.005 ^b^	0.039 ± 0.002 ^b,c^	0.041 ± 0.002 ^b,c^	D, E, DxE
(18:0)	9.292 ± 0.427 ^a,b^	9.590 ± 0.819 ^a,b^	7.355 ± 0.290 ^a^	11.173 ± 0.641 ^b^	8.270 ± 0.265 ^a^	9.059 ± 0.779 ^a,b^	E, DxE
(18:1)	1.109 ± 0.055 ^a,b^	1.024 ± 0.041 ^a^	1.038 ± 0.059 ^a^	1.364 ± 0.095 ^b^	1.077 ± 0.046 ^a^	1.280 ± 0.067 ^a,b^	D, E
(18:2)	1.061 ± 0.086 ^a^	1.138 ± 0.080 ^a^	1.014 ± 0.040 ^a^	0.743 ± 0.092 ^a,b^	0.574 ± 0.024 ^b^	0.646 ± 0.048 ^b^	D
(18:3)	0.021 ± 0.002 ^a^	0.023 ± 0.002 ^a^	0.020 ± 0.001 ^a^	0.011 ± 0.001 ^b^	0.008 ± 0.000 ^b^	0.008 ± 0.000 ^b^	D
(20:1)	0.050 ± 0.003	0.047 ± 0.004	0.039 ± 0.002	0.056 ± 0.008	0.043 ± 0.004	0.056 ± 0.004	
(20:2)	0.031 ± 0.002 ^a^	0.032 ± 0.003 ^a^	0.028 ± 0.001 ^a^	0.018 ± 0.002 ^b^	0.014 ± 0.001 ^b^	0.017 ± 0.001 ^b^	D
(20:3)	0.027 ± 0.002 ^a,c^	0.026 ± 0.001 ^a^	0.027 ± 0.003 ^a,b,c^	0.043 ± 0.004 ^b,c^	0.036 ± 0.002 ^bc^	0.040 ± 0.003 ^b^	D
(20:4)	1.123 ± 0.046 ^a,b^	1.115 ± 0.094 ^a^	1.021 ± 0.077 ^a^	1.466 ± 0.099 ^b^	1.015 ± 0.048 ^a^	1.173 ± 0.090 ^a,b^	D, E, DxE
(22:4)	0.047 ± 0.002	0.045 ± 0.003	0.044 ± 0.002	0.045 ± 0.003	0.039 ± 0.002	0.043 ± 0.002	
(22:5)	0.046 ± 0.008 ^a,b^	0.037 ± 0.003 ^a^	0.039 ± 0.004 ^a^	0.061 ± 0.005 ^b^	0.048 ± 0.006 ^a,b^	0.050 ± 0.007 ^a,b^	D
(22:6)	0.337 ± 0.030 ^a,b^	0.336 ± 0.036 ^a,b^	0.237 ± 0.011 ^a^	0.379 ± 0.025 ^b^	0.250 ± 0.018 ^a^	0.280 ± 0.037 ^a,b^	E, DxE

Diets and training sessions started in female rats after the weaning period and extended for 8 weeks. The training program was conducted 5 days per week for 30 min. The circulating levels of lysophospholipids belonging to lysophosphatidylcholine (Lyso-PC) and lysophosphatidylethanolamine (Lyso-PE) lipid subclasses were determined at the end of the study after a 12 h fast. The data are presented as means ± SEM (*n* = 9–12). The statistical comparison among the six groups of animals was conducted using two- and one-way ANOVA. D: the effect of diet; E: the effect of exercise; DxE: the interaction between the two main factors (2-way ANOVA, *p* < 0.05). ^a,b,c^: Mean values with different lowercase letters were significantly different (one-way ANOVA and Tukey or Dunnett’s T3 post hoc contrasts, *p* < 0.05).

## Data Availability

The data presented in this study are available in this article and supplementary materials.

## Supplementary Materials

The following are available online at https://www.mdpi.com/article/10.3390/metabo11080471/s1. Figure S1: Flowchart with the experimental design followed in the animal experiment, Figure S2: Standard curves used for the analysis of endogenous Lyso-PLs, Table S1: Molecular weight, transitions and optimal collision energies used for UHPLC- + ESI-MS/MS analysis of the Lyso-PLS.
