# Supplementary material for: Chronic Effect of a Cafeteria Diet and Intensity of Resistance Training on the Circulating Lysophospholipidome in Young Rats
Source: Metabolites. 2021 Jul 22;11(8):471. doi: 10.3390/metabo11080471 (PMC8398762; doi:10.3390/metabo11080471)
Supplement: Supplementary file 1 [file metabolites-11-00471-s001.zip › metabolites-1242311-supplementary.pdf]

Supplementary material

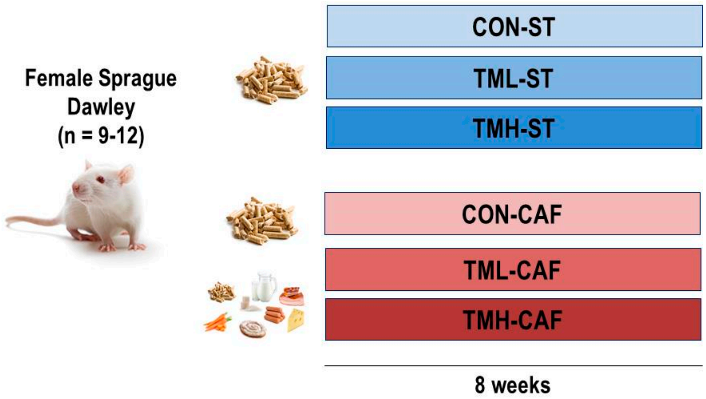

Supplementary Figure S1. Flowchart with the experimental design followed in the animal experiment. During 8 weeks, the animals were fed ad libitum either ST or CAF. Both dietary groups followed a periodic training on a treadmill at different intensities (CON: 0; TML: 12 m/min; TMH: 17 m/min). The training sessions were organized 5 days per week and extended for 30 min. At the end of the study animals were fasted overnight and sacrificed by beheading.

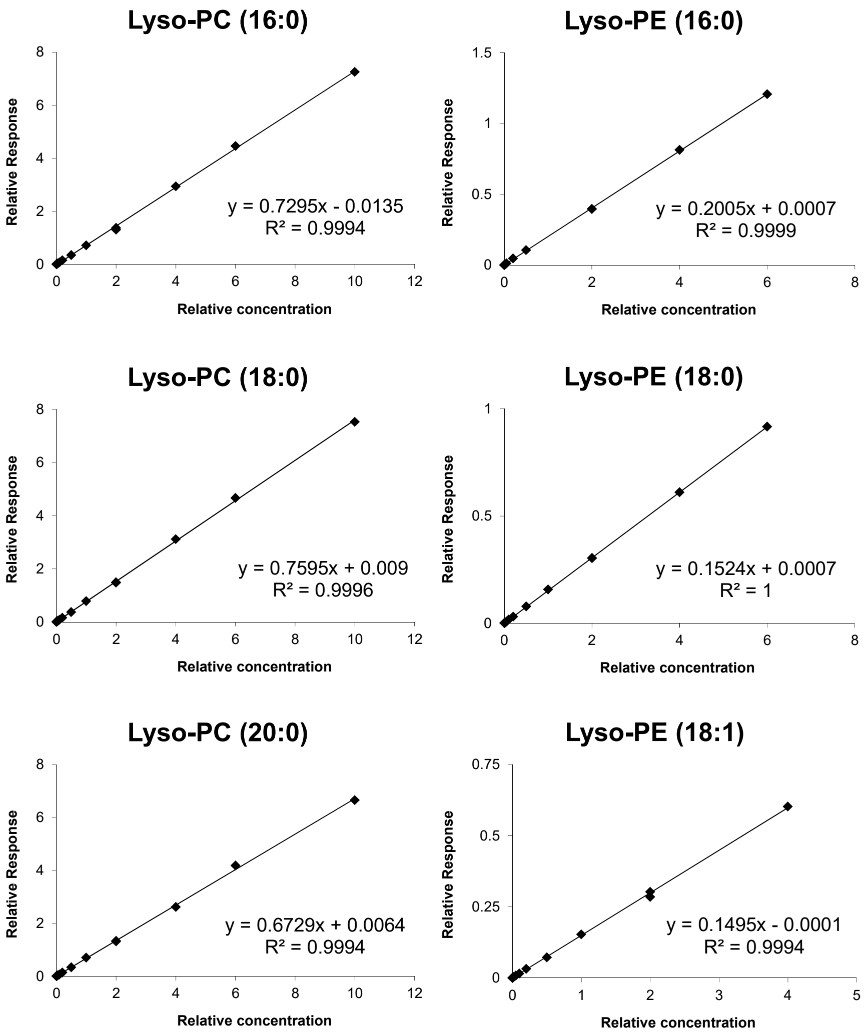

Supplementary Figure S2. Standard curves used for the analysis of endogenous Lyso-PLs.

Supplementary Table S1. Molecular weight, transitions and optimal collision energies used for UHPLC- + ESI-MS/MS analysis of the Lyso-PLS

**Metabolite**	***Molecular weight***	***Parent ion (m/z)***	***Daughter ions (m/z)***	***Collision energies (V)***
Lyso-PCs				
**(14:0)**	467.58	468.3	184.0, 104.1, 86.0	24, 28, 40
**(15:0)**	481.60	482.3	184.0, 104.1, 86.0	24, 28, 40
**(16:0)**	495.63	496.3	184.0, 104.1, 86.0	24, 28, 40
**(16:1)**	493.61	494.3	184.0, 104.1, 86.0	24, 28, 40
**(17:0)**	509.66	510.3	184.0, 104.1, 86.0	32, 28, 40
**(17:1)**	507.64	508.3	184.0, 104.1, 86.0	32, 28, 40
**(18:0)**	523.68	524.3	184.0, 104.1, 86.0	32, 28, 40

**(18:1)**	521.67	522.3	184.0, 104.1, 86.0	32, 28, 40
**(18:2)**	519.65	520.3	184.0, 104.1, 86.0	32, 28, 40
**(18:3)**	517.64	518.3	184.0, 104.1, 86.0	32, 28, 40
**(20:0)**	551.74	552.3	184.0, 104.1, 86.0	28, 28, 40
**(20:1)**	549.72	550.3	184.0, 104.1, 86.0	28, 28, 40
**(20:2)**	547.70	548.3	184.0, 104.1, 86.0	28, 28, 40
**(20:3)**	545.69	546.3	184.0, 104.1, 86.0	28, 28, 40
**(20:4)**	543.67	544.3	184.0, 104.1, 86.0	28, 28, 40
**(20:5)**	541.66	542.3	184.0, 104.1, 86.0	28, 28, 40
**(22:5)**	569.71	570.3	184.0, 104.1, 86.0	28, 28, 40
**(22:6)**	567.69	568.3	184.0, 104.1, 86.0	28, 28, 40
Lyso-PEs				
**(16:0)**	453.55	454.3	313.3, 436.3, 62.0	16, 12, 12
**(16:1)**	451.53	452.3	313.3, 436.3, 62.0	16, 12, 12
**(18:0)**	481.60	482.3	341.3, 464.3, 62.0	16, 12, 12
**(18:1)**	479.59	480.3	339.3, 462.3, 62.0	16, 12, 12
**(18:2)**	477.57	478.3	337.3, 460.3, 62.0	16, 12, 12
**(18:3)**	475.56	476.3	335.3, 458.3, 62.0	16, 12, 12
**(20:1)**	507.64	508.3	367.3, 490.3, 62.0	16, 12, 12
**(20:2)**	505.62	506.3	365.3, 488.3, 62.0	16, 12, 12
**(20:3)**	503.61	504.3	363.3, 486.3, 62.0	16, 12, 12
**(20:4)**	501.59	502.3	361.3, 484.3, 62.0	16, 12, 12
**(22:4)**	529.65	530.3	389.3, 512.3, 62.0	16, 12, 12
**(22:5)**	527.63	528.3	387.3, 510.3, 62.0	16, 12, 12
**(22:6)**	525.61	526.3	385.3, 508.3, 62.0	16, 12, 12
